# Prevalence and Regional Distribution of Beta-Hemoglobin Variants in Saudi Arabia: Insights from the National Premarital Screening Program”

**DOI:** 10.1007/s44197-024-00281-x

**Published:** 2024-07-29

**Authors:** Mansour Aljabry, Suha Sulimani, Ghazi Alotaibi, Hassan Aljabri, Shaker Alomary, Omar Aljabri, Maha Sallam, Abdulrahman Alsultan

**Affiliations:** 1 Department of Pathology, College of Medicine, King Saud University Medical City King Saud University, Riyadh, Saudi Arabia; 2 Premarital Program, Ministry of Health, Riyadh, Saudi Arabia; 3 Division of Hematology/Oncology, Department of Medicine, College of Medicine, King Saud University, Riyadh, Saudi Arabia; 4 Premarital Program Center, Madina region, Ministry of Health, Riyadh, Saudi Arabia; 5 Laboratory Department, Cluster 3 Bin Riyadh Region, Ministry of Health, Riyadh, Saudi Arabia; 6 Laboratory Department, Alahsa Cluster, Ministry of Health, Riyadh, Saudi Arabia; 7 Department of Pediatrics, College of Medicine, King Saud University, Riyadh, Saudi Arabia

**Keywords:** Genetic variants, Hemoglobinopathies, Pre-marital screening

## Abstract

**Background:**

Hemoglobinopathies are among the most prevalent inherited disorders globally, with carrier prevalence varying significantly across regions. In Saudi Arabia, high rates of consanguineous marriages amplify the risk of these disorders.

**Aim:**

This study aims to assess the burden of hemoglobinopathies by evaluating the prevalence and regional distribution of beta-hemoglobin variants, including rare variants, among couples participating in the national premarital screening program.

**Methods:**

Data were collected from the premarital genetic screening program and entered into the SEHA platform, covering the 13 administrative regions of Saudi Arabia. Blood samples underwent various screening tests for infectious and genetic diseases. Hemoglobin electrophoresis samples were analyzed using capillary electrophoresis, High-Performance Liquid Chromatography (HPLC), or a combination of both methods.

**Results:**

From 2011 to 2018, 1,871,184 individuals were included in the study, with 49.8% male and 50.2% female. The average age was 30.2 years. Hemoglobin S (HbS) was identified in 88,431 individuals (4.7% of the tested population and 78.5% of abnormal screening results), primarily as a sickle cell trait. β-thalassemia was the second most common disorder, identified in 22,420 individuals (1.2% of the population and 19.9% of hemoglobin disorders). HbC and HbD were each detected in 0.04% of cases, while HbO-Arab was identified in 0.007% and HbG in 0.006%. Hemoglobin E and hemoglobin Lepore were found to be extremely rare.

**Conclusion:**

The study demonstrates regional variation in the prevalence of hemoglobin genetic variants in Saudi Arabia. To effectively mitigate this risk, it is imperative to strengthen public education and awareness, particularly focusing on genetic screening and counseling.

## Introduction

Hemoglobinopathies are among the most prevalent inherited disorders globally, with a carrier prevalence estimated at approximately 5% of the population [1, 2]. The majority of these carriers are affected by thalassemia and sickle cell disease, exhibiting significant regional variations [3]. The distribution of other hemoglobin variants, such as hemoglobin (Hb) C, HbD, HbO-Arab, HbE, and Hb Lepore, is influenced by ethnicity and geographic location. For instance, HbC is commonly found in West Africa, where it is associated with malaria protection [4, 5]. HbD, specifically HbD-Punjab, is prevalent in Pakistan and Northwestern India [6, 7]. HbO-Arab is widespread among populations in the Middle East and North Africa, while HbE is highly prevalent in Southeast Asia [8–10].

In Saudi Arabia, hemoglobinopathies such as sickle cell disorders and thalassemia are notably common. The prevalence of the sickle cell trait ranges from 4 to 7%, and the β-thalassemia trait ranges from 1.8 to 3.2% of the population, with higher prevalence rates observed in the Eastern and Southwestern regions [11–16]. However, data on the frequency and regional distribution of less common hemoglobin variants in Saudi Arabia remain limited [8, 17, 18]. Additionally, the high rate of consanguineous marriages in Saudi Arabia, estimated at around 56%, further exacerbates the risk of these inherited disorders [19].

In response to this public health concern, the Saudi government implemented the Premarital Screening and Genetic Counseling (PMSGC) program in 2004. This initiative mandates screening for hemoglobinopathies and genetic counseling for all potential couples, aiming to support informed marital decisions and mitigate the risk of hereditary hemoglobin disorders.

This epidemiological study, the largest of its kind in Saudi Arabia, aims to evaluate the burden of hemoglobinopathies by assessing the prevalence of beta-globin variants, including rare variants, among couples undergoing the PMSGC program. Furthermore, we analyzed the regional distribution of these hemoglobin variants across the country from 2011 to 2018.

## Methods

### Study Design

This study utilized data from the Premarital Screening and Genetic Counseling (PMSGC) program for the period between 2011 and 2018. The PMSGC program systematically gathers information from individuals undergoing premarital screening and genetic counseling since 2004.

The collected data were entered into the SEHA platform, a comprehensive electronic repository encompassing the 13 administrative regions of the Kingdom of Saudi Arabia. The SEHA platform ensures the preservation of all relevant information for individuals obtaining premarital certificates. This study was approved by the Ministry of Health’s Institutional Review Board (IRB No. 23 − 18 E). All data were anonymized prior to analysis to maintain confidentiality and comply with ethical standards.

### Premarital Screening Program and Genetic Counseling (PMSGC)

The PMSGC program is supported by an extensive network of 352 healthcare reception clinics, including 270 government and 82 private clinics. Additionally, 172 affiliated laboratories and 204 genetic counseling clinics are distributed across the 13 administrative regions of Saudi Arabia. Couples seeking a marriage certificate must visit a pre-marriage center for screening. At these centers, healthcare personnel collect essential demographic data, provide necessary health education, and obtain blood samples using EDTA anticoagulant tubes. The blood samples are subjected to a battery of tests, including complete blood count, hemoglobin electrophoresis, sickling test, peripheral blood smear, reticulocyte count, and serological screenings for Hepatitis B, HIV, and Hepatitis C.

### Methods and Analysis of Hemoglobin Electrophoresis

Hemoglobin electrophoresis samples are analyzed to identify abnormal hemoglobin bands using capillary electrophoresis, High-Performance Liquid Chromatography (HPLC), or a combination of both techniques by skilled medical personnel at PMSGC centers. Laboratory specialists and physicians follow standardized protocols to interpret the test results, with complex cases being referred to the central program committee for further evaluation. The General Directorate of Health Affairs oversees the collection and management of data through regional premarital centers. All laboratory centers are accredited by the Saudi Central Board for Accreditation of Healthcare Institutions (CBAHI). Data entered into the SEHA platform are reviewed and approved by assigned physicians at each premarital center. Extracted data were organized in an Excel sheet for final statistical analysis. The prevalence of common and rare beta-hemoglobin variants was expressed per 10,000 individuals and assessed across different regions of Saudi Arabia from 2011 to 2018. The prevalence rates of HbS and β-thalassemia were compared with previously published data from the PMSGC program.

### Statistical Analysis

Continuous data were analyzed using descriptive statistics, including means, standard deviations, and frequencies for categorical variables. The association between regional distribution and prevalence was evaluated using the chi-square test. Simple linear regression was employed to assess temporal changes in the prevalence of hemoglobin disorders. A P-value of less than 0.05 was considered statistically significant. Data from the SEHA platform were consolidated, and analyses were conducted using MATLAB v.2023a (MathWorks Inc., Natick, MA, USA). Choropleth maps were generated using Datawrapper (www.datawrapper.de) to visually represent the regional distribution of hemoglobin variants.

## Results

### Prevalence and Geographic Distribution of Beta-hemoglobin Variants

**Fig. 1:**
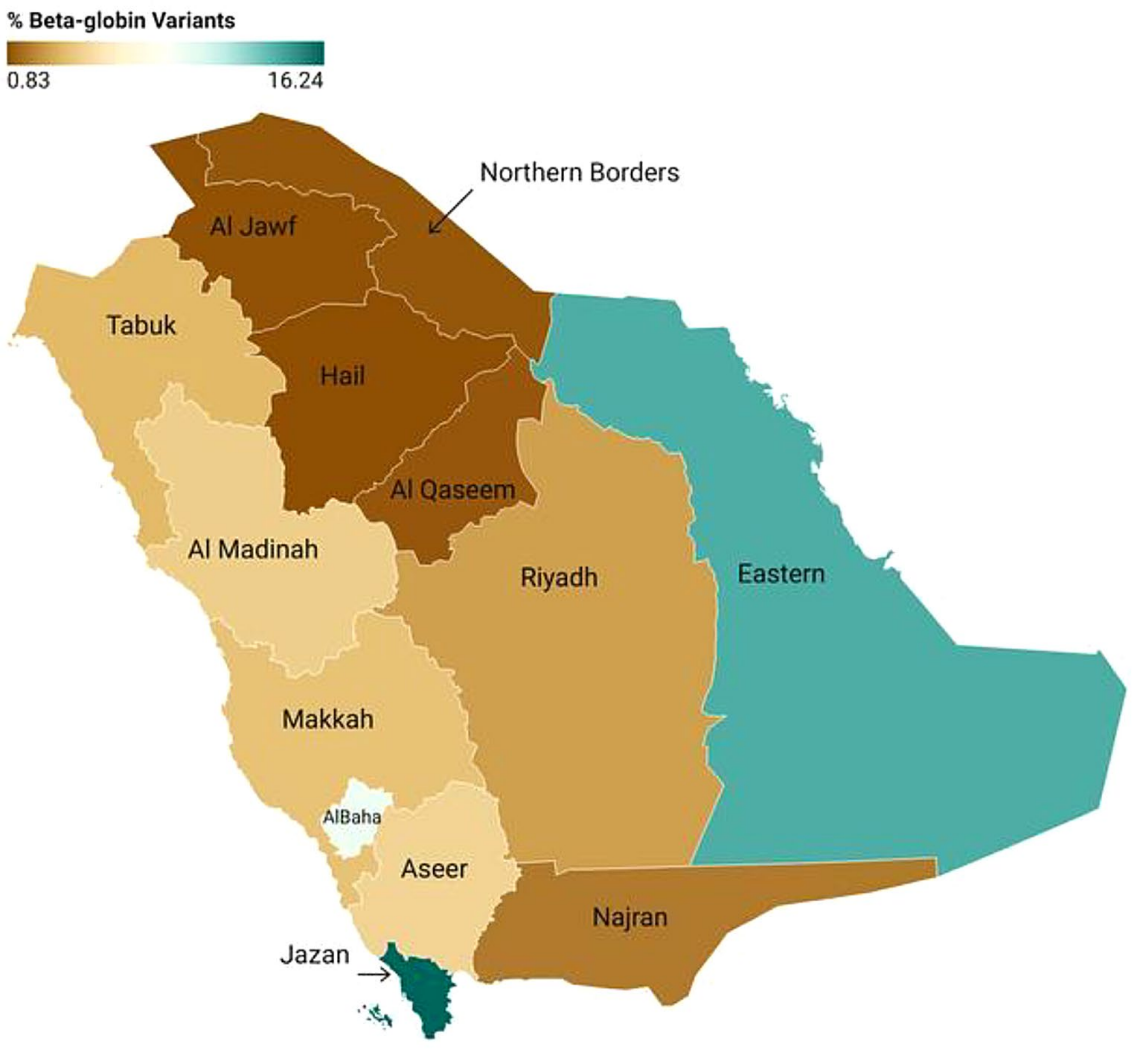
The prevalence of beta-globin variants based on the premarital screening program for hemoglobinopathies among different regions in Saudi Arabia from 2011 to 2018. Created with Data wrapper

From 2011 to 2018, a total of 1,871,184 individuals were enrolled in this study (Table 1). Of these, 49.8% were male and 50.2% were female. The mean age of the participants was 30.2 years, with a standard deviation of 8.0 years. Among the tested individuals, 112,618 (6.0%) exhibited abnormal test results, with no statistically significant difference between males and females (*P* = 0.08). Figure 1 illustrates the geographic distribution of abnormal results, revealing higher rates in regions near the Red Sea and Arabian Gulf compared to the central region. Specifically, the Eastern and Jazan regions had prevalence rates five and six times higher than Riyadh, respectively.

**Table 1:** Results of a premarital screening program for hemoglobinopathies in Saudi Arabia from 2011 to 2018

Category	Male no. (%)	Female no. (%)	Total no. (%)
**Number of Tested Individuals**	931,099	940,085	1,871,184
**Mean Age in years (± SD)**	32.8 (± 7)	27.5 (± 7)	30.2 ± 8.0
**Abnormal tests**	55,756 (6.0)	56,862 (6.0)	112,618 (6.0)
**HbS**			
HbSA	41,062 (4.4)	41,951 (4.5)	83,013 (4.4)
HbSS	2,402 (0.3)	2,326 (0.3)	4,728 (0.3)
HbS-β0/+ thalassemia	354 (0.04)	322 (0.03)	676 (0.04)
HbSC	3 (0.0003)	2 (0.0002)	5 (0.0003)
HbSD	3 (0.0003)	6 (0.0006)	9 (0.0005)
**β-thalassemia**			
Carrier State	10,443 (1.1)	11,069 (1.2)	21,512 (1.1)
β+/+, β+/0, β0/0	607 (0.07)	301 (0.03)	908 (0.05)
**HbC**			
HbCA	392 (0.04)	372 (0.04)	764 (0.04)
HbCC	5 (0.0005)	4 (0.0004)	9 (0.0005)
**HbD**			
HbDA	345 (0.04)	381 (0.04)	726 (0.04)
HbDD	8 (0.0009)	11 (0.0012)	19 (0.001)
HbO-Arab trait	59 (0.006)	67 (0.007)	126 (0.007)
HbG trait	61 (0.007)	55 (0.006)	106 (0.006)
HbE trait	9 (0.001)	4 (0.0004)	13 (0.0007)
Hb Lepore trait	3 (0.0003)	2 (0.0002)	5 (0.0003)

### Sickle Cell Hemoglobin (HbS)

HbS was identified in 88,431 individuals, primarily as the sickle cell trait. This represents 4.7% of the tested population and 78.5% of those with abnormal screening results (Table 1). There was no statistically significant gender difference in the prevalence of HbS (*P* = 0.21). Sickle cell disease predominantly presented as HbSS (87.3%), followed by HbS-β0/+ thalassemia (12.5%), and rarely as HbSC or HbSD (0.2%). The highest occurrence in the Jazan and Eastern regions, followed by the Al Baha region. Areas along the Red Sea exhibited higher rates compared to inland regions.

### β-thalassemia

β-thalassemia was the second most common hemoglobin disorder, identified in 22,420 individuals, either as a trait (96%) or disease (4%). This accounts for 1.2% of the screened population and 19.9% of individuals with hemoglobin disorders. Notably, males with β-thalassemia disease were identified twice as often as females (OR 2.03, 95% CI 1.77–2.33, *P* < 0.0001), whereas males with the β-thalassemia trait were identified less frequently than females (OR 0.95, 95% CI 0.92–0.97, *P* = 0.0003). The highest prevalence in the Jazan, Eastern, and Aseer regions. However, the Al Baha region did not exhibit a high prevalence of β-thalassemia compared to HbS.

### Rare Variants

The study also identified several rare beta-globin variants, including HbC, HbO-Arab, HbE, HbD, HbG, and Hb Lepore (Table 1). HbC was the most frequently detected variant, with 778 cases, predominantly in the Western regions of Makkah and Al Madinah. Only nine individuals were homozygous for HbC. HbD was found in 754 cases, with the highest prevalence in the Northern region of Tabuk. Nineteen individuals were homozygous for HbD. HbO-Arab was very rare, detected in 126 cases, with no homozygotes identified. HbG was found in 106 individuals, with higher prevalence in the Tabuk and Jazan regions. Carriers of HbE and Hb Lepore were extremely rare, with only 13 and 5 cases, respectively.

**Fig. 2:**
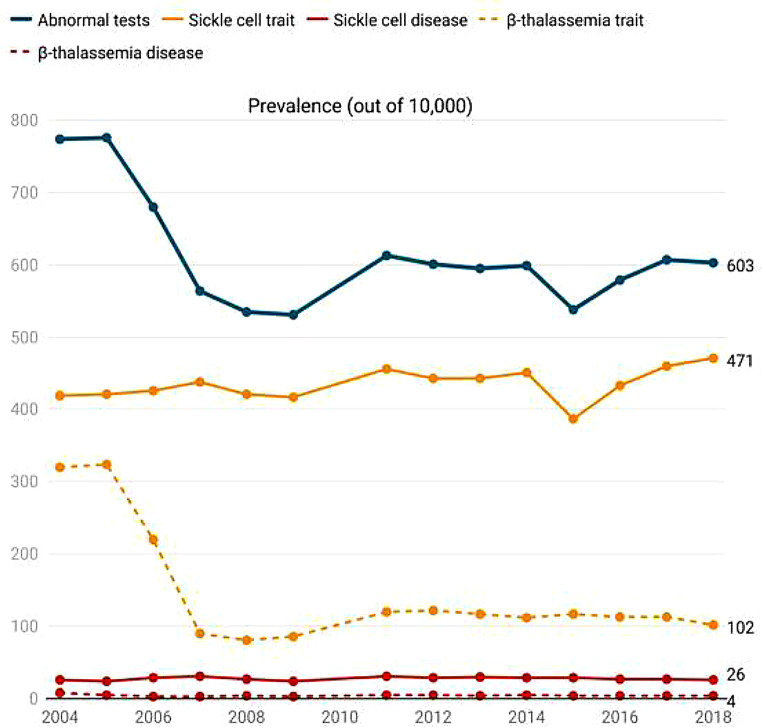
Trends in the rate of HbS and β-thalassemia in premarital screening program (2004–2018)

The rate of abnormal results decreased from nearly 7% in 2004–2006 to 5–6% in subsequent years (Fig. 2). This reduction was also observed in the rate of β-thalassemia trait during the same period, likely due to the shift from conventional hemoglobin electrophoresis techniques to HPLC and/or capillary electrophoresis, which provide more accurate HbA2 values. The trend analysis from 2007 to 2018 revealed no significant change in the rate of abnormal screening results (simple linear regression, R²=0.25, *P* = 0.11). The prevalence of sickle cell trait ranged between 387 and 471 per 10,000 screened individuals, with no significant trend change (R²=0.10, *P* = 0.33). The rate of sickle cell disease remained constant, ranging from 24 to 31 per 10,000 (R²=0.05, *P* = 0.52). The β-thalassemia trait prevalence varied between 81 and 122 per 10,000, with no notable trend variation (R²=0.34, *P* = 0.06). β-thalassemia disease was identified in only 3–5 per 10,000 (R²=0.10, *P* = 0.34).

## Discussion

The present study represents the most extensive epidemiological investigation to date on the prevalence and regional distribution of beta-hemoglobin variants in Saudi Arabia, leveraging data from the national Premarital Screening and Genetic Counseling Program (PMSGC) from 2011 to 2018. The overall prevalence of beta-globin variants was determined to be 6% of the Saudi population. This encompasses common variants such as Sickle Cell Hemoglobin (HbS) and β-thalassemia, as well as rarer variants like HbC, HbD, HbO-Arab, HbG, HbE, and Hb Lepore.

Consistent with our findings, Memish et al. (2011) reported a prevalence of 6.3% for beta-globin chain variations in a study involving 1,572,140 individuals from 2004 to 2009, with sickle cell disease and β-thalassemia affecting 4.5% and 1.8% of the population, respectively [13]. Similarly, Alhamdan et al. (2007) and by Alsaeed et al. (2018) identified comparable prevalence rates, underscoring the persistent burden of these hemoglobinopathies [11, 20].

The rate of abnormal results decreased from nearly 7% during 2004–2006 to 5–6% in subsequent years. This reduction was observed in the β-thalassemia trait rate, while the rate of sickle cell trait remained constant. This finding aligns with the studies by Memish et al. and Alsaeed et al. [13, 20] Conversely, Rao et al. (2024) reported an upward trend in β-thalassemia prevalence during the same period, noting an increase of 0.6%. [21].

Saudi Arabia has a notably higher prevalence of both the sickle cell trait and β-thalassemia trait compared to other Gulf countries, particularly in the Eastern Province. Oman has a relatively high prevalence of the sickle cell trait, while the UAE, Qatar, and Bahrain have lower prevalence rates for both conditions compared to Saudi Arabia [22–26].

Our study confirms that the prevalence of hemoglobinopathies is unevenly distributed across Saudi Arabia. Significant regional disparities exist, particularly between Hail and Jizan, with prevalence rates ranging from 0.83 to 16%. A 2011 study in Jazan, a southern region of Saudi Arabia, reported the second-highest occurrence rate of β-thalassemia in the country [21].

The highest prevalence of the sickle cell trait was observed in Jazan (135.7 per 1000), followed by the Eastern Region (114.4 per 1000). Hail exhibited the lowest prevalence (2.0 per 1000), while Al-Baha, Makkah, and Asir had intermediate rates ranging from 31 to 42 per 1000. The Eastern Region showed the highest prevalence of sickle cell disease (9.8 per 1000), with slightly lower rates in the Jazan and Asir regions (6.8–7 per 1000). Hail had the lowest incidence rate (0.1 per 1000). These regional differences suggest that environmental, genetic, and socio-cultural factors may influence the distribution of hemoglobinopathies.

Our findings underscore the importance of sustained efforts in genetic counseling and public health education. The persistent high prevalence of hemoglobinopathies in Saudi Arabia indicates that awareness and educational programs must be intensified, particularly targeting high school students and young adults. By improving understanding and awareness of genetic diseases, these programs can help reduce the incidence of sickle cell disease and beta-thalassemia in the high-risk Saudi population.

Additionally, the regional disparities in the prevalence of hemoglobinopathies suggest the need for tailored public health interventions. Strategies should consider local socio-cultural and genetic factors to effectively address the burden of these disorders. Future research should focus on identifying the underlying causes of regional differences and evaluating the long-term impact of the PMSGC program on reducing the prevalence of hemoglobinopathies.

## Conclusion and Future Prospects

This study underscores the high prevalence of hemoglobin variants in Saudi Arabia, with significant regional variability. Despite awareness of the risks, many carriers proceed with marriage. Enhanced educational programs targeting high school students about genetic screening and counseling are essential to improve genetic disease awareness and reduce the incidence of sickle cell disease and beta-thalassemia. Tailored public health interventions, considering local socio-cultural and genetic factors, are necessary to effectively address the burden of these disorders. Continued efforts in public health education, genetic counseling, and research are crucial to mitigate the impact of hemoglobinopathies in Saudi Arabia.

## Data Availability

No datasets were generated or analysed during the current study.
